# Develop a diagnostic tool for dementia using machine learning and non-imaging features

**DOI:** 10.3389/fnagi.2022.945274

**Published:** 2022-08-29

**Authors:** Huan Wang, Li Sheng, Shanhu Xu, Yu Jin, Xiaoqing Jin, Song Qiao, Qingqing Chen, Wenmin Xing, Zhenlei Zhao, Jing Yan, Genxiang Mao, Xiaogang Xu

**Affiliations:** ^1^Department of Biostatistics, The George Washington University, Washington, DC, United States; ^2^Department of Mathematics, Drexel University, Philadelphia, PA, United States; ^3^Department of Neurology, Affiliated Zhejiang Hospital, Zhejiang University School of Medicine, Zhejiang University, Hangzhou, China; ^4^Department of Radiology, Sir Run Run Shaw Hospital, Zhejiang University School of Medicine, Hangzhou, China; ^5^Zhejiang Provincial Key Lab of Geriatrics & Geriatrics Institute of Zhejiang Province, Department of Geriatrics, Affiliated Zhejiang Hospital, Zhejiang University School of Medicine, Zhejiang University, Hangzhou, China

**Keywords:** dementia, Alzheimer’s disease, early diagnostic tool, machine learning, non-imaging factors

## Abstract

**Background:**

Early identification of Alzheimer’s disease or mild cognitive impairment can help guide direct prevention and supportive treatments, improve outcomes, and reduce medical costs. Existing advanced diagnostic tools are mostly based on neuroimaging and suffer from certain problems in cost, reliability, repeatability, accessibility, ease of use, and clinical integration. To address these problems, we developed, evaluated, and implemented an early diagnostic tool using machine learning and non-imaging factors.

**Methods and results:**

A total of 654 participants aged 65 or older from the Nursing Home in Hangzhou, China were identified. Information collected from these patients includes dementia status and 70 demographic, cognitive, socioeconomic, and clinical features. Logistic regression, support vector machine (SVM), neural network, random forest, extreme gradient boosting (XGBoost), least absolute shrinkage and selection operator (LASSO), and best subset models were trained, tuned, and internally validated using a novel double cross validation algorithm and multiple evaluation metrics. The trained models were also compared and externally validated using a separate dataset with 1,100 participants from four communities in Zhejiang Province, China. The model with the best performance was then identified and implemented online with a friendly user interface. For the nursing dataset, the top three models are the neural network (AUROC = 0.9435), XGBoost (AUROC = 0.9398), and SVM with the polynomial kernel (AUROC = 0.9213). With the community dataset, the best three models are the random forest (AUROC = 0.9259), SVM with linear kernel (AUROC = 0.9282), and SVM with polynomial kernel (AUROC = 0.9213). The F1 scores and area under the precision-recall curve showed that the SVMs, neural network, and random forest were robust on the unbalanced community dataset. Overall the SVM with the polynomial kernel was found to be the best model. The LASSO and best subset models identified 17 features most relevant to dementia prediction, mostly from cognitive test results and socioeconomic characteristics.

**Conclusion:**

Our non-imaging-based diagnostic tool can effectively predict dementia outcomes. The tool can be conveniently incorporated into clinical practice. Its online implementation allows zero barriers to its use, which enhances the disease’s diagnosis, improves the quality of care, and reduces costs.

## Introduction

Dementia is a clinical syndrome of brain diseases, involving the progressive loss of memory, language, thinking and abilities of action, which seriously affects patients’ daily life and physical and mental health (Mitchell et al., 2009). The types of dementia are usually divided into Alzheimer’s disease (AD), vascular dementia (VaD), frontotemporal dementia (FTD), dementia with Lewy bodies (DLB), and other dementias, of which Alzheimer’s disease accounts for more than 60% (Geldmacher and Whitehouse, 1996; Bruun et al., 2018). Alzheimer’s disease is a neurodegenerative disease of the nervous system associated with aging. The main features of Alzheimer’s disease contain progressive memory impairment, visual-spatial ability, executive function impairments, amnesia, aphasia, apraxia, and agnosia, accompanied by personality and behavior changes. The amyloid beta (Aβ) in plaques, phosphorylated tau protein in neurofibrillary tangles are defining neuropathological features of AD (Long and Holtzman, 2019; Selkoe, 2019; Janelidze et al., 2020). With aging, the prevalence and incidence of Alzheimer’s disease are increasing rapidly every year, which brings a heavy burden to patients, their families, and social and economic development (Rabins et al., 1982; Azevedo et al., 2021). Cognitive impairment that does not meet dementia criteria refers to mild cognitive impairment (MCI), which is consistently shown to have a high risk of progression to dementia (Petersen et al., 1999; Grundman et al., 2004). Unlike Alzheimer’s disease patients, MCI patients have no significant effect on activities of daily work and life (Feldman et al., 2006).

Timely and accurate diagnosis of dementia is the key to the prevention and treatment of dementia. At present, the main methods used for dementia and MCI detection include clinical screening of scales, pathological tissue biopsy, and medical imaging diagnosis. Clinical screening of scales is the most common detection method used in clinical practice. This method mainly involves interactive communication between professionals and patients or patients’ family members. By objectively and comprehensively collecting information from multiple perspectives, clinical screening of scales can help make effective evaluation judgments (Sheehan, 2012; Bissig and DeCarli, 2019). Nevertheless, this method relies on the objectivity of data collection and the professional level of evaluators, and can therefore be time-consuming and expensive. Another method used in clinical and scientific studies is pathological tissue biopsy, which mainly examines biomarkers in the cerebrospinal fluid such as Aβ and Tau protein. Brain biopsies are rarely performed in clinics. The disadvantage of this approach is that its invasiveness and potential risk of complications can have a certain negative impact on the patient’s health. In addition, even if the biopsy is negative, it does not completely rule out the possibility of dementia, because there may be lesions in other parts of the brain (Warren et al., 2005; Josephson et al., 2007; Leinonen et al., 2010). The third diagnostic method uses medical images, such as functional PET/MR. As an auxiliary diagnostic method, medical imaging is useful in many cases, e.g., to rule out the cognitive decline caused by secondary tumors or stroke. This method also has many limitations. First, given the high cost of equipment, not all hospitals can be equipped with corresponding testing equipment. Second, the cost of testing imposes a heavy financial burden to patients. Third, but not least, since the morphological and pathological changes in molecules and tissues may not be obvious at the early stage of the disease, imaging diagnosis often has blind spots in detection (Mosconi, 2013; Ishii, 2014; Barthel et al., 2015; Shivamurthy et al., 2015).

To assist and improve traditional dementia diagnosis methods, machine learning and deep learning have been increasingly applied in AD detection, especially in classifying neuroimaging data (Pellegrini et al., 2018; Jo et al., 2019). Frequently used machine learning algorithms to classify AD neuroimages include support vector machines (SVM) and artificial neural networks (ANN) (Pellegrini et al., 2018). With the development of deep learning techniques, the performance of deep learning models have in general surpassed that of machine learning in classifying neuroimaging data and have become the dominant method for dealing with such data. At present, the convolutional neural network (CNN) is the most widely used deep learning architecture for the diagnostic classification of AD, due to its effectiveness in dealing with imaging data (Jo et al., 2019). Compared with the CNN models trained from scratch in earlier years (Sarraf and Tofighi, 2016), most recently developed CNN models have incorporated transfer learning to make better use of small-scale datasets for better model performance (Hon and Khan, 2017; Islam and Zhang, 2017; Aderghal et al., 2018; Ding et al., 2019). The dimensions of the neural images CNN can process have also been upgraded from two to three (Hosseini-Asl et al., 2016; Khvostikov et al., 2018). In addition to CNN, some other important deep learning architectures have also been actively studied for AD diagnosis, including deep belief networks (Cai et al., 2016), deep auto-encoder (Dolph et al., 2017; Shi et al., 2017), recurrent neural network (Cui et al., 2019; Lee et al., 2019), etc.

Although neuroimaging based machine learning/deep learning algorithms have achieved a high accuracy in many AD classification tasks, these algorithms are not always the best path to build the automated AD diagnosis system for a number of reasons. First, training neuroimaging based models typically requires a large amount of high-quality labeled medical imaging, which can be a huge challenge because of the institutional barriers and the cost of collecting and labeling data. Second, due to the limitation of design, the image-based models typically cannot utilize other types of data that may contain specific information for prediction. Third, due to geographic, economic or other constraints, patients may not be able to access the established models or provide the image required by the model. Fourth, due to the complex structure or inherent “black-box” limitation, it is difficult to understand the relationship between selected variables and predicted results or the relative importance of each selected feature, resulting in difficulties in interpreting models and correcting potential biases arising from the training data. These problems can greatly limit models’ clinical relevance, practical application value, and the possibility of future improvement. Integration of non-imaging features (such as patient demographics, cognitive test results, clinical covariates, etc.) has been called to further advance the field of AD diagnosis (Pellegrini et al., 2018).

In this study, we explored the diagnostic effects of non-imaging features from the nursing home located in Hangzhou, Zhejiang Province, China. We employed a wide range of advanced machine learning models and validated these models in two ways: internally using a novel double cross-validation (CV) algorithm (Krstajic et al., 2014) and externally on a separate dataset from four communities in Zhejiang. Apart from developing predictive models, we analyzed the features that are important to the diagnosis of AD/MCI and discussed their roles in clinical practice. We also used the best-performing model to build an automated AD/MCI detection tool online. This study shows that non-imaging factors can be exploited to obtain rich predictive information and create good diagnostic models for AD/MCI. In the literature there are few studies on machine learning with non-imaging features to detect AD/MCI. Shankle et al. (1997) used decision tree learners, rule learners, and the Naive Bayesian classifier on the non-imaging dataset from University of California to learn the best decision rules to distinguish normal brain aging from the earliest stages of dementia. Maroco et al. (2011) compared 10 machine learning algorithms using several neuropsychological tests as predictors for predicting the evolution into dementia in elderly people with MCI. We note that the clinical questions investigated in these studies are different from the clinical question we studied, and that they lacked adequate internal/external valuation or online implementation. Therefore our research adds to the knowledge in this area.

## Materials and methods

### Data

**FIGURE 1:**
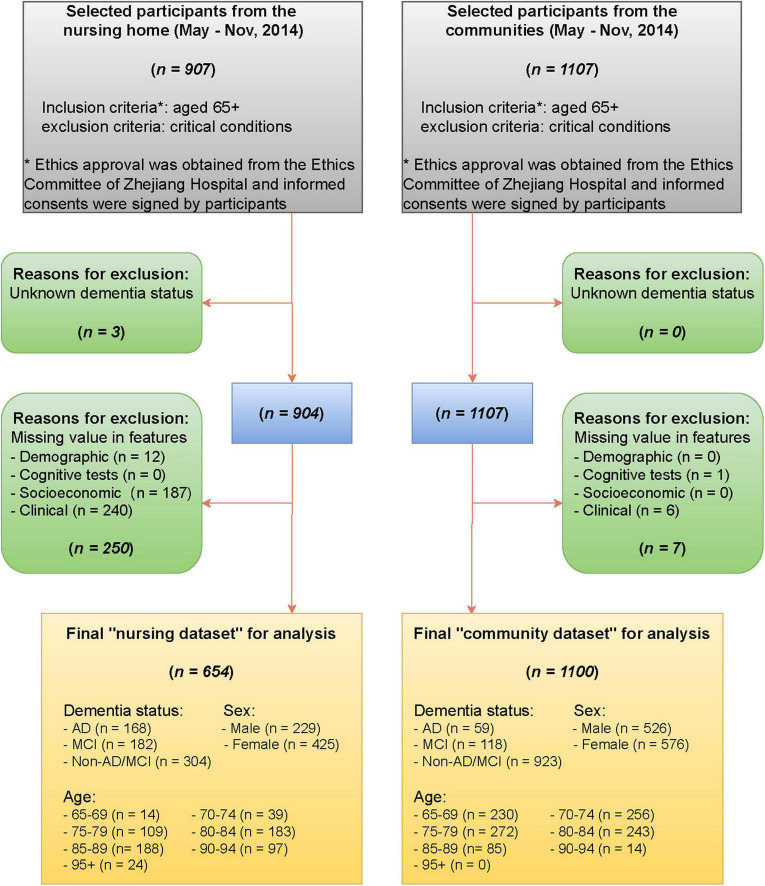
Flow diagram of selecting participants from the nursing home and the communities.

The data were collected from the nursing home in Hangzhou, Zhejiang Province, China, between May and November 2014. Patients aged 65 or older were included and those in critical conditions were excluded. The data contain dementia status and 70 non-imaging features derived from demographic information (sex, age), cognitive tests [Mini-Cog test, clock drawing test (CDT), Mini-Mental State Exam (MMSE), and AD8 screening], socioeconomic information (education, main occupation before retirement, marital status), and clinic characteristics (past medical history, smoking status, alcohol status). Detailed definitions and demographic characteristics of the 70 features are listed in Supplementary Tables 1, 2, respectively. The dementia status of patients were evaluated by experienced physicians based on the combination of the clinical diagnostic criteria for Alzheimer’s disease, the Clinical Dementia Rating (CDR) scale, and reports from patients’ families about the patients’ daily life (e.g., memory of the way home, memory of past life, response to usual communication, etc.), and classified into three levels AD, MCI, non-AD/MCI (Albert et al., 2011; McKhann et al., 2011). To facilitate the analysis, we removed observations with missing values in cognitive status or any of the 70 features. The final nursing dataset for analysis includes 654 patients. See Figure 1 for a detailed data collection process.

**FIGURE 2:**
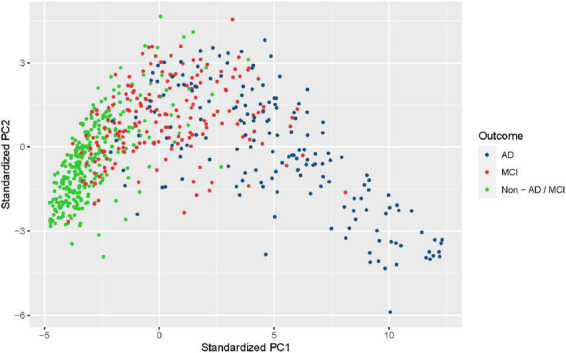
Visualization of the nursing dataset after dimension reduction with principal component analysis. Black dots represent patients diagnosed with Alzheimer’s disease (AD), red dots represent patients diagnosed with mild cognitive impairment (MCI), and green dots represent patients from neither class (non-AD/MCI).

The nursing data were collected from the nursing home and hence were based on a population with a high risk of AD/MCI. In the final nursing dataset, 168 participants (25.7%) were diagnosed with AD, 182 (27.8%) with MCI, and 304 (46.5%) with Non-AD/MCI. It is seen that observed outcomes were roughly balanced for two classes (53.5% AD/MCI vs. 46.5% normal). Principal component analysis, a dimension reduction technique, was performed to reduce the nursing data into two-dimensional and visualize it (Figure 2). The clusters of AD and non-AD/MCI barely overlap, while the cluster of MCI is mixed with the other two classes. This shows that three classes can present a clear pattern through non-imaging features.

For validation purposes, we collected data with the same cognitive status and non-imaging features from four communities across 12 counties in Zhejiang province. A multi-stage stratified random cluster sampling method was adopted to select the communities. We first divided 12 counties into four groups based on economic levels, then systematically selected one district from each of these four groups, and finally randomly chose one community from each district. The same inclusion/exclusion criteria and data processing progress as for the nursing dataset were applied (Figure 1). The final community dataset contains 1,100 cases, of which 59 (5.4%) were diagnosed with AD, 118 (10.7%) with MCI, and 923 (83.9%) with non-AD/MCI.

For the collection of the nursing and community data, ethics approval was obtained from the Ethics Committee of Zhejiang Hospital and informed consents were signed by participants.

### Machine learning methods

#### Logistic regression

The logistic regression (Hosmer et al., 2013) is a classical machine learning method widely used in medical research. It performs well if there is a linear relationship between the features and logit of the outcome. Due to the simplicity of the design, inferences regarding the contribution of each feature in predicting the outcome can be easily drawn from the model. We used this simple but powerful classification model as a benchmark: the included machine learning models should have at least as good predictive performance as the logistic model.

#### Support vector machines

The SVM (Vapnik, 2013), a state-of-the-art machine learning algorithm, is a generalization of linear decision boundaries for classification. It has successful applications in a variety of medical classification tasks, e.g., diagnosing heart valve diseases (Comak et al., 2007), breast cancer (Akay, 2009), diabetes (Yu et al., 2010), etc. Using a kernel function, SVM can transform the data into a higher-dimensional space and construct a linear boundary in the new space while generating a non-linear boundary in the original space. In this study, we tried four different SVM models SVM_l, SVM_r, SVM_s, and SVM_p, which correspond to models obtained by using linear, radial basis, sigmoid, and polynomial kernel functions, respectively.

#### Neural networks

Neural networks are non-linear machine learning models with computing systems inspired by how the human brain processes information. They have been widely applied in image analysis, biochemical analysis, drug design, diagnostic systems, and other branches of human medicine (Amato et al., 2013). Despite the low interpretability, neural networks are very flexible and quite effective in purely predictive settings thanks to the model-free design. In this study, we used a feedforward neural network with a single layer of hidden neurons and L1 weight regularization, which can be thought of as a non-linear generalization of linear logistic regression. The single-layer design and weight regularization can constrain the complexity of the model and prevent it from overfitting.

#### Random forests

Random forests use de-correlated tree predictors to build powerful classification models (Breiman, 2001). By combining predictions from a large collection of individual decision trees, random forests outperform the individual tree predictor and produce more accurate predictions. Being easy to understand and effective to use, random forests have many applications in the medical field, such as predicting Alzheimer’s and other diseases (Khalilia et al., 2011; Gray et al., 2013; Kane et al., 2014). In the random forest, a small number of trees will lead to poor performance of the model while a large number will not cause the model to overfit (Hastie et al., 2001). Therefore, we used a large number of trees, 500 in this study, to build the random forest model. In the model training process, we also assessed the importance of each feature by computing the mean decrease in Gini Index (Louppe et al., 2013).

#### XGBoosting

Boosting uses a combination of many “weak” classifiers to generate an ultimate strong classifier (Hastie et al., 2001). Though less popular than SVMs and random forests, boosting can provide outstanding prediction performance (Zhang et al., 2017). One popular boosting algorithm is gradient boosted decision trees (GBDT) (Friedman, 2002), which use the decision tree as the weak learner and use the gradient descent algorithm to minimize the loss function of the model. In this paper, we implemented XGBoost (short for “Extreme Gradient Boosting”), one of the most efficient implementations of GBDT (Chen and Guestrin, 2016). Compared to GBDT, XGBoost achieves better performance by introducing the regularization term in the loss function (the model is trained by minimizing the loss function) and using a second-order Taylor approximation for the loss function.

#### The least absolute shrinkage and selection operator

To better understand the relationship between features and outcome variables, we employed the least absolute shrinkage and selection operator (LASSO) (Tibshirani, 1996), which allows automatic feature selection by adding an L1 regularization term to force coefficients of some features to be equal to zero. LASSO is a popular and arguably the most effective method for selecting features in a linear model (Hastie et al., 2017). It is especially useful when there is a need to analyze a large number of features. We implemented LASSO as a prediction model for AD/MCI. And we investigated the contribution of features to the outcome prediction by examining the coefficient paths of the model.

#### Best subset

Best subset selection is another approach to finding the most relevant features to predict the outcome of interest. By definition, it requires evaluating possible subsets of the collection of all features according to some criteria. Although this approach has unmatched advantages in terms of interpretability, it is computationally demanding when the number of features is large and is computationally infeasible when cross-validation is used to evaluate subsets. In this study, we used a two-step search strategy to approximate the optimal result of best subset selection and reduce the computational requirement: (1) for each *S* = 1,2,…,*P*, find the “best” *S* out of *P* features, where the best features here refer to the ones that can maximize the likelihood function of the logistic regression model (achieved by the “BeSS.ONE” function in the BeSS R package; Wen et al., 2017), (2) find the best number of *S* (achieved by regarding *S* as a parameter and tuning it with double CV).

### Evaluation metrics and validation methods

We performed both internal and external validation for all proposed machine learning models. The performance of all models was then assessed and compared according to sensitivity (recall), specificity, accuracy, area under the receiver operating characteristic curve (AUROC), precision, F1-score, and area under the precision-recall curve (AUPRC). Note that AUROC summarizes the trade-off between sensitivity and specificity while the F1-score (harmonic mean of the precision and recall) and AUPRC summarize the trade-off between sensitivity and precision.

**FIGURE 3:**
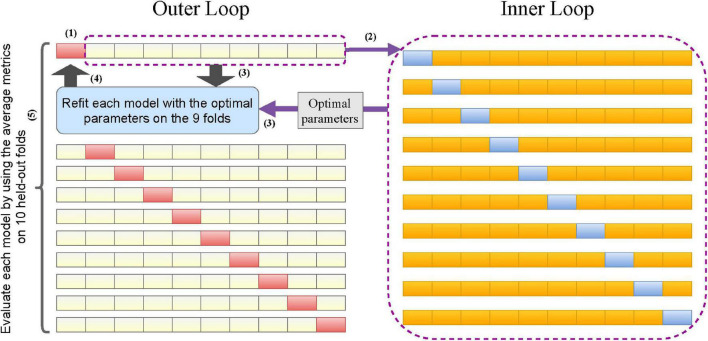
Flowchart of the double cross-validation.

We trained and internally evaluated our proposed machine learning models on the nursing dataset. The nursing dataset (350 cases) was relatively small compared to the 70 features and complex machine learning models we selected. Splitting the dataset into training, validation, and test sets allows parameter tuning. However, this method does not yield reliable estimates because of the small test set. On the other hand, the traditional *k*-fold CV can lead to over-optimistic estimates of the model’s performance (Varma and Simon, 2006). Therefore, we considered a novel double CV that can take care of both model evaluation and parameter tuning (Krstajic et al., 2014). To perform the double CV, we divided the nursing dataset into 10 folds and performed the following for each model. (1) Left one fold for validation. (2) On the remaining 9 folds, performed a 10-fold CV to tune the parameters so that the optimal parameters maximized the average AUROC. (3) Used the optimal parameters to refit the model on the 9-folds. (4) Computed evaluation metrics for the model fitted in step (3) on the held-out fold in step (1). (5) Repeated steps (1)–(4) 10 times across the 10 folds of the nursing dataset to obtain the 10 sets of evaluation metrics and optimal parameters. The performance of each model evaluated internally on the nursing dataset was then represented by the average of 10 sets of evaluation metrics, i.e., the double CV metrics. The process of performing double CV is illustrated in Figure 3.

After internal validation, we performed an external validation for the models. The medians of 10 sets of optimal parameters from the double CV were used to train the final models on the whole nursing dataset. Then the evaluation metrics of each final model were computed on the community dataset.

### Parameter optimization

In the inner loop of double CV, different parameter tuning methods were applied to different models. Logistic regression has no parameters to be tuned. Grid search was used in double CV to fine-tune the parameters in the four SVM models, neural network, random forest, LASSO, and best subset model. For the last two models, tuning parameters (L1 regularization parameter in LASSO and number of features “*S*” in the best subset) is effectively equivalent to performing feature selection. The XGBoost model contains many important parameters (e.g., learning rate, maximum depth of a tree, number of trees, etc.) and requires extensive tuning to reach the desired performance. To address this, we used the Gaussian process upper confidence bound (GP-UCB) algorithm (Srinivas et al., 2009) in the double CV to tune the parameters. GP-UCB is a popular Bayesian optimization algorithm that chooses parameters by controlling the exploration-exploitation tradeoffs.

### Software

All statistical analyses were done in R (R version 3.6.2) using the following libraries: e1071, randomForest, nnet, NeuralNetTools, xgboost, rBayesianOptimization, glmnet, BeSS, ROCR, cutpointr, ggplot2, and their respective dependencies. The online screening tool was employed by using R Shiny.

## Results

### Models’ evaluation and comparison

We have applied a number of different machine learning algorithms to build prediction models for detecting AD/MCI. It is of interest to compare these methods and determine which one performed best.

**FIGURE 4:**
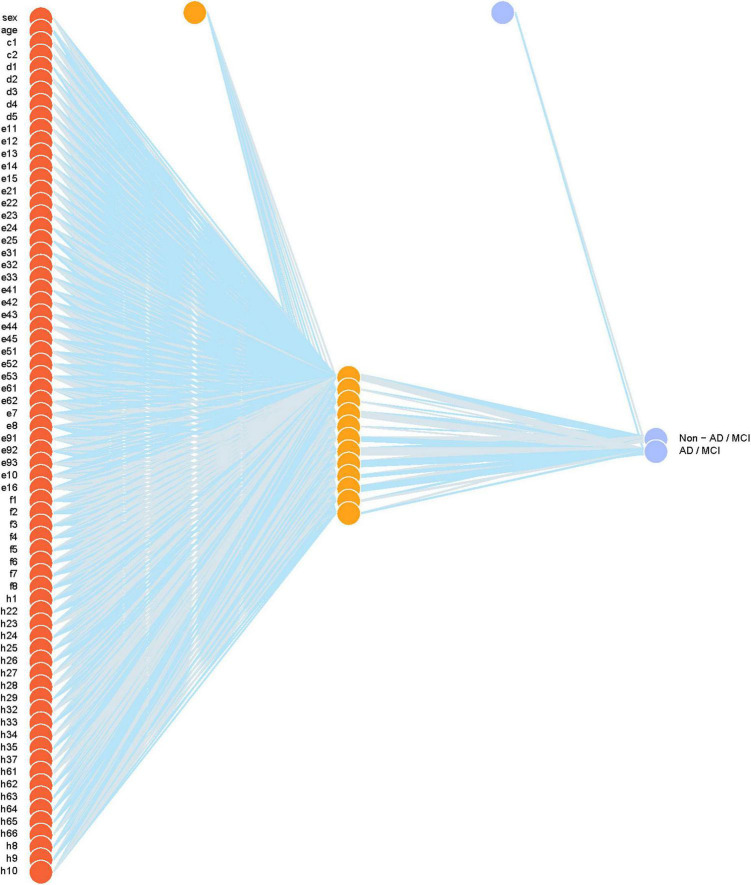
The final neural network model. Weights are color-coded by sign (blue+, gray−) and thickness is in proportion to magnitude. Input features include sex, age, Mini-Cog test (c1–c2), Clock Drawing test (d1–d5), Mini-Mental State exam (e11–e15, e21–e25, e31–e33, e41–e45, e51–e53, e61–e62, e7, e8, e91–e93, e10, e16), AD8 screening (f1–f8), education (h1), occupation (h22–h29), marital status (h32–h35, h37), past medical history (h61–66), number of medications (h8), smoking (h9), and drinking (h10).

Table 1 compares demographic characteristics of age, sex, education, and summary scores of cognitive tests across the normal, MCI, and AD groups. Table 2 reports the classification performance of each model evaluated internally by the double CV on the nursing dataset. The averages of the 10 values of sensitivity, specificity, accuracy, and AUROC in the outer loop of double CV were provided. It can be seen that the SVM_r model had the best sensitivity while the SVM_p had the best specificity. The SVM_p and LASSO had the best accuracy performance. The Neural network (Figure 4) achieved the best AUROC, followed by XGBoost and SVM_p.

**TABLE 1:** Demographic characteristics of age, sex, education, and summary scores of cognitive tests across the normal, MCI, and AD groups.

	Nursing dataset				Community dataset			
	AD	MCI	Non-AD/MCI	Overall	AD	MCI	Non-AD/MCI	Overall
	(*N* = 168)	(*N* = 182)	(*N* = 304)	(*N* = 654)	(*N* = 59)	(*N* = 118)	(*N* = 923)	(*N* = 1,100)
**Sex**								
Male	52 (31.0%)	63 (34.6%)	114 (37.5%)	229 (35.0%)	30 (50.8%)	48 (40.7%)	448 (48.5%)	526 (47.8%)
Female	116 (69.0%)	119 (65.4%)	190 (62.5%)	425 (65.0%)	29 (49.2%)	70 (59.3%)	475 (51.5%)	574 (52.2%)
**Age** (SD)	85 (± 6.4)	85 (± 5.6)	82 (± 6.4)	84 (± 6.3)	80 (± 5.4)	79 (± 5.7)	75 (± 6.2)	76 (± 6.3)
**Education** (SD)	2.3 (± 1.3)	2.7 (± 1.3)	3.4 (± 1.4)	2.9 (± 1.4)	2.4 (± 1.2)	2.9 (± 1.1)	3.4 (± 1.2)	3.3 (± 1.2)
**Summary score of mini-cog test** (SD)	1.0 (± 1.1)	2.1 (± 1.2)	3.2 (± 0.88)	2.3 (± 1.4)	2.1 (± 1.2)	2.6 (± 1.2)	3.6 (± 0.69)	3.4 (± 0.90)
**Summary score of clock drawing test** (SD)	0.98 (± 1.4)	2.4 (± 1.8)	4.2 (± 1.3)	2.9 (± 2.0)	3.1 (± 1.6)	3.9 (± 1.3)	4.7 (± 0.70)	4.6 (± 0.95)
**Summary score of mini-mental state exam** (SD)	13 (± 6.6)	22 (± 4.3)	27 (± 2.6)	22 (± 7.2)	21 (± 4.7)	24 (± 3.8)	28 (± 2.2)	28 (± 3.2)
**Summary score of AD8 screening** (SD)	4.3 (± 2.6)	2.1 (± 1.8)	0.80 (± 1.1)	2.1 (± 2.3)	4.3 (± 2.2)	3.8 (± 2.0)	1.7 (± 1.1)	2.0 (± 1.5)

**TABLE 2:** Classification performance of each model evaluated internally by the double CV on the nursing dataset.

Method	Sensitivity	Specitivity	Accuracy	AUROC
Logistic regression	0.8229	0.8289	0.8256	0.9068
SVM_l	0.8143	0.8453	0.8287	0.9127
SVM_r	0.8600	0.8455	0.8532	0.9287
SVM_s	0.8200	0.8976	0.8562	0.9374
SVM_p	0.8343	0.8947	0.8624	0.9378
Neural network	0.8429	0.8751	0.8578	0.9435
Random forest	0.8314	0.8618	0.8455	0.9340
XGBoost	0.8457	0.8552	0.8501	0.9398
LASSO	0.8400	0.8882	0.8624	0.9341
Best subset	0.8371	0.8553	0.8456	0.9141

Table 3 reports the classification performance of each model evaluated externally on the community dataset. Compared to the nursing dataset, the community data were collected at households and were based on a population with a low risk of AD/MCI. The observed outcomes were quite unbalanced between the two classes (16.1% AD/MCI vs. 83.9% normal). Therefore, in addition to sensitivity, specificity, accuracy, and AUROC, we recorded the metrics of precision, F1-score, and AUPRC as these metrics are especially useful for the unbalanced data. From Table 3, we can see that the SVM_r and SVM_p have the best accuracy. SVM_l, SVM_p, and random forest models are the top three models in terms of AUROC (> 0.92). The SVM_r and SVM_p models had the highest F1-scores while logistic regression, XGBoost, and best subset models had poor F1-scores. The AUPRC values of SVM models, neural network, and random forest were relatively high, while those of the remaining models were relatively low.

**TABLE 3:** Classification performance of each model evaluated externally on the community dataset.

Method	Sensitivity	Specitivity	Accuracy	AUROC	Precision	F1-score	AUPRC
Logistic regression	0.6215	0.8895	0.8464	0.8435	0.5189	0.5656	0.5199
SVM_l	0.5763	0.9458	0.8864	0.9282	0.6711	0.6201	0.6652
SVM_r	0.6102	0.9404	0.8873	0.9137	0.6626	0.6353	0.6395
SVM_s	0.5650	0.9437	0.8827	0.9177	0.6579	0.6079	0.6560
SVM_p	0.6045	0.9415	0.8873	0.9213	0.6646	0.6331	0.6549
Neural network	0.5876	0.9426	0.8855	0.9139	0.6624	0.6228	0.6513
Random forest	0.5706	0.9437	0.8836	0.9259	0.6601	0.6121	0.6623
XGBoost	0.5424	0.9415	0.8773	0.9006	0.6400	0.5872	0.6323
LASSO	0.5932	0.9393	0.8836	0.9023	0.6522	0.6213	0.6284
Best subset	0.4859	0.9274	0.8564	0.8483	0.5621	0.5212	0.5432

Though, according to Tables 2, 3, no model appears to be superior to all other models by all evaluation metrics, we can find some clues based on the accuracy and AUROC. The accuracy is the most common and intuitive metric to compare models’ performance. SVM_p and LASSO have the best accuracy in the internal validation (balanced data) while SVM_r and SVM_p have the best accuracy in the external validation (imbalanced data). According to this metric, the SVM and LASSO models seem to be the best models. However, we must note that accuracies have flaws in comparing models’ overall performance. First, the metric is limited to a single decision threshold (0.5 in this study), with which the model’s prediction probability is compared to determine whether the outcome prediction is positive or negative. Second, the metric cannot be used to compare models that are built upon datasets with different outcome distributions. Third, the metric can be misleading on classification problems with a skewed class distribution. For example, the accuracy on a dataset with 10% positive cases can be at least 90% accurate.

**FIGURE 5:**
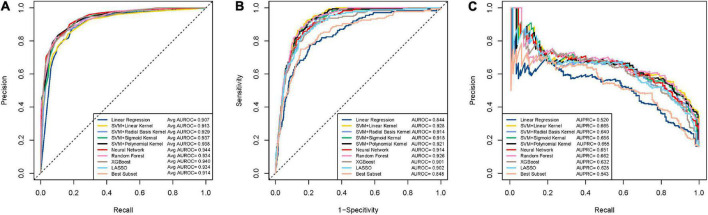
ROC and PRC curves for the machine learning models for detecting dementia. **(A)** Mean ROC curves for the models on the nursing datasets. Each curve represents the mean ROC curve from the outer loop of the double cross validation. **(B)** ROC curves for the models on the community data. **(C)** PRC curves for the models on the community data.

Compared to the accuracy, AUROC is a more robust and informative evaluation metric as it summarizes the sensitivity and specificity across different decision thresholds and can be used to compare models on different (possibly skewed) outcome distributions. On the nursing dataset, the neural network, Xgboost, SVM_p have the highest AUROC values (Figure 5A). However, the neural network and Xgboost have a low AUROC on the community dataset (Figure 5B). On the community dataset, the SVM_l, random forest, and SVM_p were the top models in terms of AUROC, while the first two models had less performance than the third model on the nursing dataset. It can be seen that SVM_p is the only one performing well on both the nursing (balanced) and the community (unbalanced) datasets.

While ROC can be used for skewed outcome distributions, it may mask poor performance under unbalanced data (Jeni et al., 2013). F1 score and AUPRC, two metrics based on precision, are useful for the unbalanced setting since precision is an informative measure under imbalanced data (Saito and Rehmsmeier, 2015). We examined F1-score and AUPRC on the community dataset to rule out underperforming models on these two metrics. It can be seen that SVM_p has relatively high scores on these two measures (Figure 5C), which again confirms that SVM_p is robust on the unbalanced data. Through the above analysis, we concluded that overall SVM_p is the best AD/MCI detection model. We had implemented SVM_p online for patients and healthcare professionals to access (R Shiny).^1^

In terms of AUROC, our SVM_p is one of the best-performing models in predicting dementia to date (Hou et al., 2019). While most published models relied on a small number of features and few reported (proper) validation results (Pellegrini et al., 2018; Hou et al., 2019), our models took advantage of 70 features and went through both internal (double cross) validation and external validation.

Therefore, our study made maximum use of the information from available features while ensuring the reliability and repeatability of the results.

### Features’ contribution

Some of the machine learning models we built enabled us to see the contribution of the features for predicting AD/MCI. We studied the relative importance of features by the tree-based random forests and XGBoost models and further investigated the role of the important features by the Lasso model and the best subset model.

In the random forest model, the Gini index is used to decide which feature to split at each node within each component tree classifier. Each split of a node results in a decrease in Gini where the magnitude of the decrease indicates the discriminatory power of the split. For each feature, the decrease in the Gini index is accumulated each time the feature is selected to split a node. Therefore, the average decrease over all component tree classifiers for each feature can be used to represent the contribution of this feature to the prediction and thus serve as a measure of importance. A greater mean decrease means higher importance. Based on information gain, a measure similar to the mean decrease in Gini, the XGBoost can also compute each feature’s contribution to the model using all the splits.

Supplementary Figure 1A presents the 20 most important features based on the final random forest model. It can be seen that the tests of counting down from 100 by 7 (e41-e45) and clocking drawing (d1-d5) are of most importance for predicting outcome. Mini-Cog test (c1-c2), time orientation test (e11-e15), and age have a lower level of importance. Of less importance is the test of copy drawing (e16), the test of repeating phrase (e7), the test of repeating previous items (e51-e53), medications (h8), and others. The feature importance rankings given by the XGBoost model are similar to those given by the random forest (Supplementary Figure 1B), while the former highlights the importance of education (h1), and AD8 Dementia Screening (f1-f8), and the test of saying a complete sentence (e10).

To understand the specific impact of important features, we also studied the features selected by the LASSO and best subset model and analyzed the signs of the features’ coefficients. In the final LASSO model, the L1 regularization shrunk the coefficients of less important features to zero. There were 35 features considered by LASSO to be relevant to outcome prediction (Supplementary Figures 2A,B). In contrast, the best subset model selected 19 features (Supplementary Figure 2C), almost all of which are included in the features selected by LASSO. These results show that many non-imaging features could help predict AD/MCI.

Specifically, 17 were selected by both LASSO and best subset. These features were most likely to be relevant to AD/MCI prediction, including age, Mini-Cog test (c2), clocking drawing test (d2, d5), time orientation test (e15), address orientation test (e23), the test of counting down from 100 by 7 (e43, e44), the test of repeating previous items (e51, e52), the test of repeating previous items (e7), the test of saying a complete sentence (e10), AD8 Dementia Screening (f6, f8), education (h1), “cadres staff” in the occupation (h24), and “married” in the marital status (h32). For these 17 features, LASSO and the best subset agreed on the signs of their coefficients. These signs allow us to further understand the influence of the features on predicting the disease. From Supplementary Figure 2B, it can be seen that the coefficients of all test-related features were negative, which is reasonable since passing the test indicates a lower likelihood of developing AD/MCI. “Married” (compared to “single”) in the marital status also suggests a lower risk. On the other hand, age, “handling complex personal financial matters” (f6) and “daily memory and thinking” (f8) in AD8 screening, education, and “cadres staff” (compared to “worker”) in the occupation (h24) have positive coefficients, suggesting that these features are risk indicators.

**FIGURE 6:**
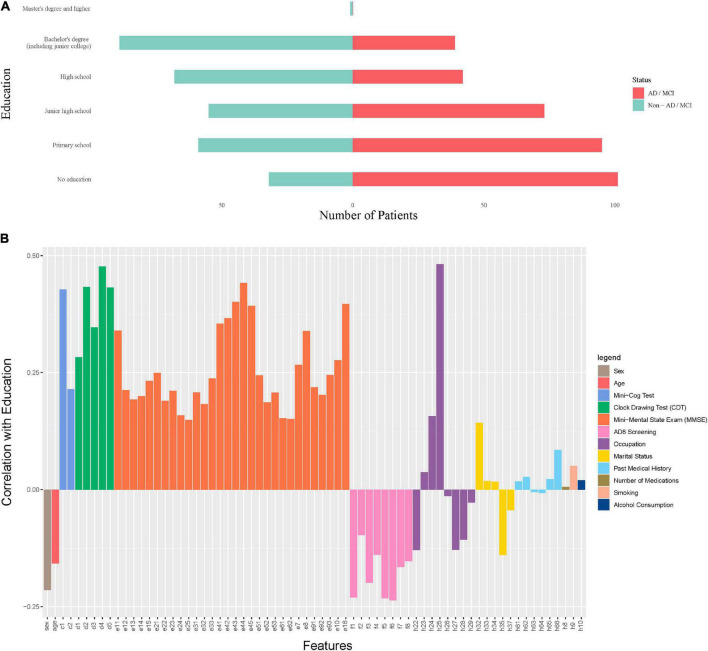
The role of education in predicting dementia. **(A)** The marginal distribution of the levels of education on the nursing dataset. **(B)** The correlation coefficients between education and other features on the nursing dataset.

In the nursing dataset, if we only look at education without considering other features, then education is negatively associated with ADI/MCI (Figure 6A). But this relationship is reversed when other features are taken into account (Supplementary Figures 2B,C). These results of education echo the findings that the role of education can be controversial in predicting the risk of dementia (Caamaño-Isorna et al., 2006; Sharp and Gatz, 2011). Instead of being an independent risk factor, education could be a factor that protects against or delays the clinical manifestations of dementia. In other words, patients with a severe brain damage and a high educational level may present similar clinical symptoms of the disease as those with a less severe brain damage and a lower educational level. This view can also be confirmed by the results in Figure 6B where education is highly positively correlated with cognitive abilities (Mini-Cog, Clock Drawing, MMSE) and negatively correlated with cognitive problems (AD8 Screening features). Therefore, our model in fact suggests that a higher educational level may mean more severe brain damage if cognitive levels are equal.

## Discussion

We created and validated our AD/MCI diagnostic tool using extensive data collected from the nursing home and the communities. These data are readily available and closely related to clinical diagnosis, which makes our model highly applicable. The applicability of our tool is further enhanced by its online deployment. Patients and clinicians who do not understand machine learning algorithms can easily input the collected non-imaging features into our tool to obtain timely predictive results. Compared to the traditional clinical screening of scales, our approach simplifies the data collection task and quickly performs high-quality analysis, thus greatly improving the diagnostic efficiency of clinicians. In addition, our approach does not involve any risk of complications associated with invasive biopsies, nor rely on equipment and operators for medical imaging systems. These merits make our approach almost free of test costs or risk of use, therefore avoiding economic or health impacts on patients and their families. We believe it has great potential applications, especially in small cities and rural areas.

## Conclusion

Automated diagnostic tools have become crucial in the diagnosis of dementia. Although neuro-images have been heavily used in recently developed tools, traditional non-imaging features can effectively diagnose dementia and conveniently incorporate clinical practice. We used a large number of non-imaging features and machine learning to create a highly performing dementia diagnostic tool. Our work leverages the predictive potential of non-imaging features and significantly lowers the barriers for using the diagnostic tool. We believe this study will have a direct impact on physicians’ diagnostic practice and patients’ self-screening.

## Data availability statement

The original contributions presented in this study are included in the article/Supplementary material, further inquiries can be directed to the corresponding authors.

## Author contributions

XX, HW, JY, and GM conceived and designed the study. SX, YJ, XJ, and SQ collected the data. HW, XX, LS, and QC performed data analysis. HW, XX, WX, and ZZ made the figures. HW and XX wrote the manuscript. All authors reviewed, approved the final version of the manuscript, and agreed to be accountable for the content of the work.

## Footnotes

1
https://hwmachinelearning.shinyapps.io/Dementia/

## Abbreviations

MCI: mild cognitive impairment
Aβ: amyloid beta
VaD: vascular dementia
FTD: frontotemporal dementia
DLB: dementia with Lewy bodies
SVM: support vector machine
XGBoost: extreme gradient boosting
LASSO: least absolute shrinkage and selection operator
ANN: artificial neural networks
CV: cross-validation
CDT: clock drawing test
MMSE: Mini-Mental State Exam
CDR: clinical dementia rating
GBDT: gradient boosted decision trees
AUROC: area under the receiver operating characteristic curve
AUPRC: area under the precision-recall curve
GP-UCB: Gaussian process upper confidence bound.
