# Supplementary material for: Develop a diagnostic tool for dementia using machine learning and non-imaging features
Source: Front Aging Neurosci. 2022 Aug 29;14:945274. doi: 10.3389/fnagi.2022.945274 (PMC9461143; doi:10.3389/fnagi.2022.945274)
Supplement: Supplementary file 1 [file Data_Sheet_1.docx]

**SUPPLEMENTARY FIGURES**


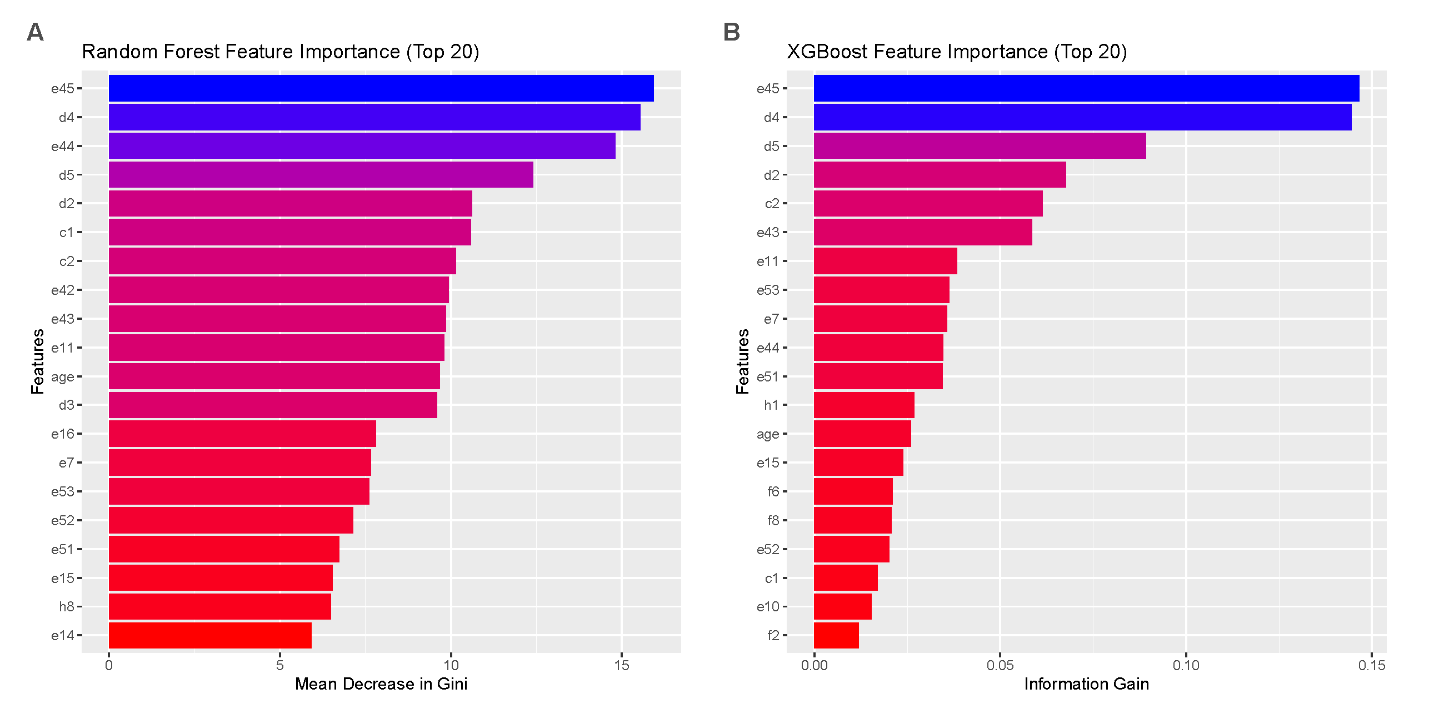
**Supplementary Fig. 1** **Feature importance plots from the random forest and XGBoost modelling.** (A) 20 most important features selected by the final random forest model. (B) 20 most important features selected by the final XGBoost model. Involved features include age, Mini-Cog test (c1-c2), Clock Drawing test (d1-d5), Mini-Mental State exam (e11-e15, e21-e25, e31-e33, e41-e45, e51-e53, e61-e62, e7, e8, e91-e93, e10, e16), AD8 screening (f1-f8), education (h1), and number of medications (h8).


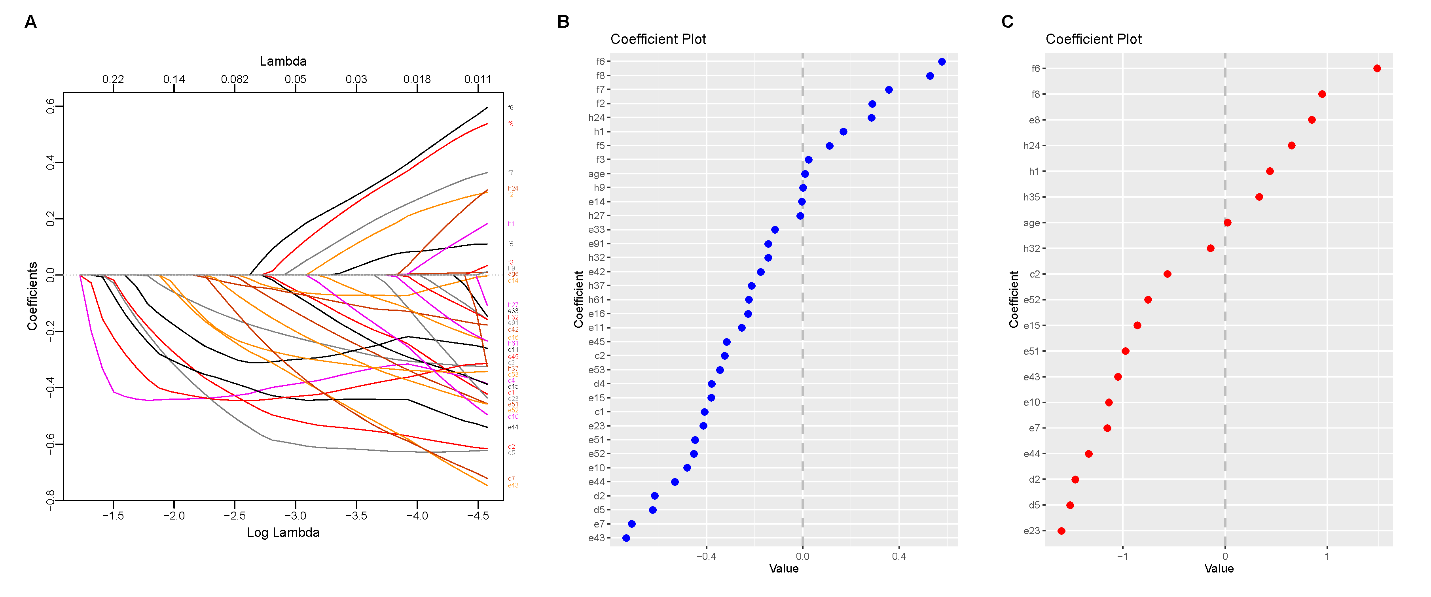


**Supplementary Fig. 2** **Coefficient plots from the final LASSO and the best subset models.** (**A**) The profile plot for the coefficients from the final LASSO model. (**B**) The dot plot for the coefficients from the final LASSO model. (**C**) The dot plot for the coefficients from the final best subset model. Involved features include age, Mini-Cog test (c1-c2), Clock Drawing test (d1-d5), Mini-Mental State exam (e11-e15, e21-e25, e31-e33, e41-e45, e51-e53, e61-e62, e7, e8, e91-e93, e10, e16), AD8 screening (f1-f8), education (h1), occupation (h22-h29), marital status (h32-h35, h37), past medical history (h61-66), and smoking (h9).

**Table S1 Definitions of the features used to build machine learning models**

| **Label** | **Feature** |
| --- | --- |
| ***Demographic*** | |
| Sex | Sex |
| Age | Age |
| ***Mini-Cog Test*** | |
| c1 | Draws a simple clock correctly |
| c2 | Number of words correctly recalled (range: 0-3) |
| ***Clock Drawing Test (CDT)*** | |
| d1 | Draws closed circle |
| d2 | Places numbers in the correct positions |
| d3 | Includes all 12 correct numbers |
| d4 | Draws two and only two hands |
| d5 | Places hands in correct positions |
| ***Mini-Mental State Exam (MMSE)*** | |
| e11 | Temporal orientation: what is the year? |
| e12 | Temporal orientation: what is the season? |
| e13 | Temporal orientation: what is the month? |
| e14 | Temporal orientation: what is the date? |
| e15 | Temporal orientation: what day of the week is it? |
| e21 | Spatial orientation: in which province are we? |
| e22 | Spatial orientation: in which city are we? |
| e23 | Spatial orientation: in which district do you live? |
| e24 | Spatial orientation: what is this place? |
| e25 | Spatial orientation: on which floor are we? |
| e31 | The examiner names three objects (ball, flag, tree) and asks the patient to repeat: the patient correctly repeats the first object |
| e32 | The examiner names three objects (ball, flag, tree) and asks the patient to repeat: the patient repeats the second object |
| e33 | The examiner names three objects (ball, flag, tree) and asks the patient to repeat: the patient repeats the third object |
| e41 | Counts down from 100 by sevens: the patient’s first subtraction is correct |
| e42 | Counts down from 100 by sevens: the patient’s second subtraction is correct |
| e43 | Counts down from 100 by sevens: the patient’s third subtraction is correct |
| e44 | Counts down from 100 by sevens: the patient’s fourth subtraction is correct |
| e45 | Counts down from 100 by sevens: the patient’s fifth subtraction is correct |
| e51 | Recalls the three objects learned earlier: the patient correctly recalls the first 3object |
| e52 | Recalls the three objects learned earlier: the patient correctly recalls the second object |
| e53 | Recalls the three objects learned earlier: the patient correctly recalls the third object |
| e61 | Names two objects (pen, watch): the patient correctly names the first object |
| e62 | Names two objects (pen, watch): the patient correctly names the second object |
| e7 | Repeats the phrase “Forty-four Stone Lions” |
| e8 | Reads the instruction “close your eyes” and obeys |
| e91 | Follows the first of a three-stage command: “take the paper in your right hand” |
| e92 | Follows the second of a three-stage command: “fold the paper in half” |
| e93 | Follows the third of a three-stage command: “put the paper on the floor” |
| e10 | Makes up and says a complete sentence that make sense |
| e16 | Copies the diagram |
| ***AD8 Screening*** | |
| f1 | Problems with judgment |
| f2 | Less interest in hobbies/activities |
| f3 | Repeats the same things over and over |
| f4 | Trouble learning how to use a tool, appliance, or gadget |
| f5 | Forgets correct month or year |
| f6 | Trouble handling complicated financial affairs |
| f7 | Trouble remembering appointments |
| f8 | Daily problems with thinking and/or memory |
| ***Socioeconomic*** | |
| h1 | Education（1 = no education, 2 = primary school, 3 = junior high school, 4 = high school, 5 = high school, 6 = bachelor’s degree (including junior college), 6 = master’s degree, 7 = doctoral degree） |
| h21 | Main occupation before retirement = worker |
| h22 | Main occupation before retirement = agricultural laborer |
| h23 | Main occupation before retirement = military personnel |
| h24 | Main occupation before retirement = cadres staff |
| h25 | Main occupation before retirement = scientific/medical/educational worker |
| h26 | Main occupation before retirement = self-employed worker |
| h27 | Main occupation before retirement = housewife |
| h28 | Main occupation before retirement = unemploymentee |
| h29 | Main occupation before retirement = unknown |
| h31 | Marital status = single |
| h32 | Marital status = married |
| h33 | Marital status = separated |
| h34 | Marital status = divorced |
| h35 | Marital status = widowed |
| h37 | Marital status = unknown |
| ***Clinical*** | |
| h61 | Past Medical History (PMH): hypertension |
| h62 | Past Medical History (PMH): diabetes |
| h63 | Past Medical History (PMH): coronary heart disease |
| h64 | Past Medical History (PMH): stroke |
| h65 | Past Medical History (PMH): cerebral trauma |
| h66 | Past Medical History (PMH): hypothyroidism |
| h8 | Number of currently taking prescription drugs |
| h9 | Smoking status (1 = never, 2 = former, 3 = current) |
| h10 | Alcohol status (1 = drinks alcohol, 0 = doesn’t drink) |

1. The category of “unmarried cohabitation” (h36) for marital status was not included because no patients fell into this category.
2. The reference category h21 can be represented by other categories (i.e., h21 is true if h22-h29 are all false) and hence is not used in the model building and analysis. The same applies to the reference category h31, which is true if h32-h37 are all false.

**Table S2** **Demographic characteristics of the features used to build machine learning models**

| **Label** | **Nursing Dataset** | | | | **Community Dataset** | | | |
| --- | --- | --- | --- | --- | --- | --- | --- | --- |
|  | **AD** | **MCI** | **Non-AD/MCI** | **Overall** | **AD** | **MCI** | **Non-AD/MCI** | **Overall** |
|  | **(N=168)** | **(N=182)** | **(N=304)** | **(N=654)** | **(N=59)** | **(N=118)** | **(N=923)** | **(N=1100)** |
| Sex |  |  |  |  |  |  |  |  |
| Male | 52 (31.0 %) | 63 (34.6 %) | 114 (37.5 %) | 229 (35.0 %) | 30 (50.8 %) | 48 (40.7 %) | 448 (48.5 %) | 526 (47.8 %) |
| Female | 116 (69.0 %) | 119 (65.4 %) | 190 (62.5 %) | 425 (65.0 %) | 29 (49.2 %) | 70 (59.3 %) | 475 (51.5 %) | 574 (52.2 %) |
| Age |  |  |  |  |  |  |  |  |
| Mean (SD) | 85 (± 6.4) | 85 (± 5.6) | 82 (± 6.4) | 84 (± 6.3) | 80 (± 5.4) | 79 (± 5.7) | 75 (± 6.2) | 76 (± 6.3) |
| c1 |  |  |  |  |  |  |  |  |
| No | 159 (94.6 %) | 150 (82.4 %) | 116 (38.2 %) | 425 (65.0 %) | 43 (72.9 %) | 64 (54.2 %) | 147 (15.9 %) | 254 (23.1 %) |
| Yes | 9 (5.4 %) | 32 (17.6 %) | 188 (61.8 %) | 229 (35.0 %) | 16 (27.1 %) | 54 (45.8 %) | 776 (84.1 %) | 846 (76.9 %) |
| c2 |  |  |  |  |  |  |  |  |
| Mean (SD) | 0.96 (± 1.1) | 1.9 (± 1.1) | 2.6 (± 0.76) | 2.0 (± 1.2) | 1.9 (± 0.99) | 2.1 (± 0.95) | 2.7 (± 0.55) | 2.6 (± 0.68) |
| d1 |  |  |  |  |  |  |  |  |
| No | 86 (51.2 %) | 31 (17.0 %) | 11 (3.6 %) | 128 (19.6 %) | 5 (8.5 %) | 12 (10.2 %) | 29 (3.1 %) | 46 (4.2 %) |
| Yes | 82 (48.8 %) | 151 (83.0 %) | 293 (96.4 %) | 526 (80.4 %) | 54 (91.5 %) | 106 (89.8 %) | 894 (96.9 %) | 1054 (95.8 %) |
| d2 |  |  |  |  |  |  |  |  |
| No | 150 (89.3 %) | 115 (63.2 %) | 60 (19.7 %) | 325 (49.7 %) | 20 (33.9 %) | 24 (20.3 %) | 35 (3.8 %) | 79 (7.2 %) |
| Yes | 18 (10.7 %) | 67 (36.8 %) | 244 (80.3 %) | 329 (50.3 %) | 39 (66.1 %) | 94 (79.7 %) | 888 (96.2 %) | 1021 (92.8 %) |
| d3 |  |  |  |  |  |  |  |  |
| No | 135 (80.4 %) | 91 (50.0 %) | 42 (13.8 %) | 268 (41.0 %) | 14 (23.7 %) | 12 (10.2 %) | 21 (2.3 %) | 47 (4.3 %) |
| Yes | 33 (19.6 %) | 91 (50.0 %) | 262 (86.2 %) | 386 (59.0 %) | 45 (76.3 %) | 106 (89.8 %) | 902 (97.7 %) | 1053 (95.7 %) |
| d4 |  |  |  |  |  |  |  |  |
| No | 147 (87.5 %) | 107 (58.8 %) | 42 (13.8 %) | 296 (45.3 %) | 41 (69.5 %) | 50 (42.4 %) | 83 (9.0 %) | 174 (15.8 %) |
| Yes | 21 (12.5 %) | 75 (41.2 %) | 262 (86.2 %) | 358 (54.7 %) | 18 (30.5 %) | 68 (57.6 %) | 840 (91.0 %) | 926 (84.2 %) |
| d5 |  |  |  |  |  |  |  |  |
| No | 158 (94.0 %) | 137 (75.3 %) | 88 (28.9 %) | 383 (58.6 %) | 33 (55.9 %) | 34 (28.8 %) | 69 (7.5 %) | 136 (12.4 %) |
| Yes | 10 (6.0 %) | 45 (24.7 %) | 216 (71.1 %) | 271 (41.4 %) | 26 (44.1 %) | 84 (71.2 %) | 854 (92.5 %) | 964 (87.6 %) |
| e11 |  |  |  |  |  |  |  |  |
| No | 128 (76.2 %) | 54 (29.7 %) | 12 (3.9 %) | 194 (29.7 %) | 13 (22.0 %) | 17 (14.4 %) | 20 (2.2 %) | 50 (4.5 %) |
| Yes | 40 (23.8 %) | 128 (70.3 %) | 292 (96.1 %) | 460 (70.3 %) | 46 (78.0 %) | 101 (85.6 %) | 903 (97.8 %) | 1050 (95.5 %) |
| e12 |  |  |  |  |  |  |  |  |
| No | 71 (42.3 %) | 5 (2.7 %) | 2 (0.7 %) | 78 (11.9 %) | 13 (22.0 %) | 7 (5.9 %) | 2 (0.2 %) | 22 (2.0 %) |
| Yes | 97 (57.7 %) | 177 (97.3 %) | 302 (99.3 %) | 576 (88.1 %) | 46 (78.0 %) | 111 (94.1 %) | 921 (99.8 %) | 1078 (98.0 %) |
| e13 |  |  |  |  |  |  |  |  |
| No | 93 (55.4 %) | 27 (14.8 %) | 9 (3.0 %) | 129 (19.7 %) | 21 (35.6 %) | 12 (10.2 %) | 7 (0.8 %) | 40 (3.6 %) |
| Yes | 75 (44.6 %) | 155 (85.2 %) | 295 (97.0 %) | 525 (80.3 %) | 38 (64.4 %) | 106 (89.8 %) | 916 (99.2 %) | 1060 (96.4 %) |
| e14 |  |  |  |  |  |  |  |  |
| No | 127 (75.6 %) | 54 (29.7 %) | 25 (8.2 %) | 206 (31.5 %) | 24 (40.7 %) | 19 (16.1 %) | 19 (2.1 %) | 62 (5.6 %) |
| Yes | 41 (24.4 %) | 128 (70.3 %) | 279 (91.8 %) | 448 (68.5 %) | 35 (59.3 %) | 99 (83.9 %) | 904 (97.9 %) | 1038 (94.4 %) |
| e15 |  |  |  |  |  |  |  |  |
| No | 117 (69.6 %) | 48 (26.4 %) | 19 (6.2 %) | 184 (28.1 %) | 24 (40.7 %) | 24 (20.3 %) | 33 (3.6 %) | 81 (7.4 %) |
| Yes | 51 (30.4 %) | 134 (73.6 %) | 285 (93.8 %) | 470 (71.9 %) | 35 (59.3 %) | 94 (79.7 %) | 890 (96.4 %) | 1019 (92.6 %) |
| e21 |  |  |  |  |  |  |  |  |
| No | 52 (31.0 %) | 8 (4.4 %) | 1 (0.3 %) | 61 (9.3 %) | 2 (3.4 %) | 1 (0.8 %) | 1 (0.1 %) | 4 (0.4 %) |
| Yes | 116 (69.0 %) | 174 (95.6 %) | 303 (99.7 %) | 593 (90.7 %) | 57 (96.6 %) | 117 (99.2 %) | 922 (99.9 %) | 1096 (99.6 %) |
| e22 |  |  |  |  |  |  |  |  |
| No | 53 (31.5 %) | 5 (2.7 %) | 1 (0.3 %) | 59 (9.0 %) | 2 (3.4 %) | 1 (0.8 %) | 1 (0.1 %) | 4 (0.4 %) |
| Yes | 115 (68.5 %) | 177 (97.3 %) | 303 (99.7 %) | 595 (91.0 %) | 57 (96.6 %) | 117 (99.2 %) | 922 (99.9 %) | 1096 (99.6 %) |
| e23 |  |  |  |  |  |  |  |  |
| No | 68 (40.5 %) | 23 (12.6 %) | 6 (2.0 %) | 97 (14.8 %) | 7 (11.9 %) | 4 (3.4 %) | 10 (1.1 %) | 21 (1.9 %) |
| Yes | 100 (59.5 %) | 159 (87.4 %) | 298 (98.0 %) | 557 (85.2 %) | 52 (88.1 %) | 114 (96.6 %) | 913 (98.9 %) | 1079 (98.1 %) |
| e24 |  |  |  |  |  |  |  |  |
| No | 62 (36.9 %) | 14 (7.7 %) | 3 (1.0 %) | 79 (12.1 %) | 9 (15.3 %) | 8 (6.8 %) | 4 (0.4 %) | 21 (1.9 %) |
| Yes | 106 (63.1 %) | 168 (92.3 %) | 301 (99.0 %) | 575 (87.9 %) | 50 (84.7 %) | 110 (93.2 %) | 919 (99.6 %) | 1079 (98.1 %) |
| e25 |  |  |  |  |  |  |  |  |
| No | 60 (35.7 %) | 8 (4.4 %) | 2 (0.7 %) | 70 (10.7 %) | 10 (16.9 %) | 24 (20.3 %) | 23 (2.5 %) | 57 (5.2 %) |
| Yes | 108 (64.3 %) | 174 (95.6 %) | 302 (99.3 %) | 584 (89.3 %) | 49 (83.1 %) | 94 (79.7 %) | 900 (97.5 %) | 1043 (94.8 %) |
| e31 |  |  |  |  |  |  |  |  |
| No | 62 (36.9 %) | 24 (13.2 %) | 11 (3.6 %) | 97 (14.8 %) | 4 (6.8 %) | 3 (2.5 %) | 2 (0.2 %) | 9 (0.8 %) |
| Yes | 106 (63.1 %) | 158 (86.8 %) | 293 (96.4 %) | 557 (85.2 %) | 55 (93.2 %) | 115 (97.5 %) | 921 (99.8 %) | 1091 (99.2 %) |
| e32 |  |  |  |  |  |  |  |  |
| No | 66 (39.3 %) | 27 (14.8 %) | 11 (3.6 %) | 104 (15.9 %) | 7 (11.9 %) | 16 (13.6 %) | 26 (2.8 %) | 49 (4.5 %) |
| Yes | 102 (60.7 %) | 155 (85.2 %) | 293 (96.4 %) | 550 (84.1 %) | 52 (88.1 %) | 102 (86.4 %) | 897 (97.2 %) | 1051 (95.5 %) |
| e33 |  |  |  |  |  |  |  |  |
| No | 60 (35.7 %) | 22 (12.1 %) | 6 (2.0 %) | 88 (13.5 %) | 9 (15.3 %) | 28 (23.7 %) | 26 (2.8 %) | 63 (5.7 %) |
| Yes | 108 (64.3 %) | 160 (87.9 %) | 298 (98.0 %) | 566 (86.5 %) | 50 (84.7 %) | 90 (76.3 %) | 897 (97.2 %) | 1037 (94.3 %) |
| e41 |  |  |  |  |  |  |  |  |
| No | 77 (45.8 %) | 19 (10.4 %) | 4 (1.3 %) | 100 (15.3 %) | 9 (15.3 %) | 3 (2.5 %) | 2 (0.2 %) | 14 (1.3 %) |
| Yes | 91 (54.2 %) | 163 (89.6 %) | 300 (98.7 %) | 554 (84.7 %) | 50 (84.7 %) | 115 (97.5 %) | 921 (99.8 %) | 1086 (98.7 %) |
| e42 |  |  |  |  |  |  |  |  |
| No | 139 (82.7 %) | 84 (46.2 %) | 40 (13.2 %) | 263 (40.2 %) | 28 (47.5 %) | 38 (32.2 %) | 72 (7.8 %) | 138 (12.5 %) |
| Yes | 29 (17.3 %) | 98 (53.8 %) | 264 (86.8 %) | 391 (59.8 %) | 31 (52.5 %) | 80 (67.8 %) | 851 (92.2 %) | 962 (87.5 %) |
| e43 |  |  |  |  |  |  |  |  |
| No | 144 (85.7 %) | 98 (53.8 %) | 53 (17.4 %) | 295 (45.1 %) | 30 (50.8 %) | 48 (40.7 %) | 89 (9.6 %) | 167 (15.2 %) |
| Yes | 24 (14.3 %) | 84 (46.2 %) | 251 (82.6 %) | 359 (54.9 %) | 29 (49.2 %) | 70 (59.3 %) | 834 (90.4 %) | 933 (84.8 %) |
| e44 |  |  |  |  |  |  |  |  |
| No | 148 (88.1 %) | 111 (61.0 %) | 55 (18.1 %) | 314 (48.0 %) | 41 (69.5 %) | 53 (44.9 %) | 114 (12.4 %) | 208 (18.9 %) |
| Yes | 20 (11.9 %) | 71 (39.0 %) | 249 (81.9 %) | 340 (52.0 %) | 18 (30.5 %) | 65 (55.1 %) | 809 (87.6 %) | 892 (81.1 %) |
| e45 |  |  |  |  |  |  |  |  |
| No | 154 (91.7 %) | 118 (64.8 %) | 64 (21.1 %) | 336 (51.4 %) | 42 (71.2 %) | 58 (49.2 %) | 137 (14.8 %) | 237 (21.5 %) |
| Yes | 14 (8.3 %) | 64 (35.2 %) | 240 (78.9 %) | 318 (48.6 %) | 17 (28.8 %) | 60 (50.8 %) | 786 (85.2 %) | 863 (78.5 %) |
| e51 |  |  |  |  |  |  |  |  |
| No | 138 (82.1 %) | 88 (48.4 %) | 65 (21.4 %) | 291 (44.5 %) | 20 (33.9 %) | 26 (22.0 %) | 92 (10.0 %) | 138 (12.5 %) |
| Yes | 30 (17.9 %) | 94 (51.6 %) | 239 (78.6 %) | 363 (55.5 %) | 39 (66.1 %) | 92 (78.0 %) | 831 (90.0 %) | 962 (87.5 %) |
| e52 |  |  |  |  |  |  |  |  |
| No | 153 (91.1 %) | 117 (64.3 %) | 110 (36.2 %) | 380 (58.1 %) | 43 (72.9 %) | 52 (44.1 %) | 221 (23.9 %) | 316 (28.7 %) |
| Yes | 15 (8.9 %) | 65 (35.7 %) | 194 (63.8 %) | 274 (41.9 %) | 16 (27.1 %) | 66 (55.9 %) | 702 (76.1 %) | 784 (71.3 %) |
| e53 |  |  |  |  |  |  |  |  |
| No | 145 (86.3 %) | 108 (59.3 %) | 81 (26.6 %) | 334 (51.1 %) | 43 (72.9 %) | 69 (58.5 %) | 222 (24.1 %) | 334 (30.4 %) |
| Yes | 23 (13.7 %) | 74 (40.7 %) | 223 (73.4 %) | 320 (48.9 %) | 16 (27.1 %) | 49 (41.5 %) | 701 (75.9 %) | 766 (69.6 %) |
| e61 |  |  |  |  |  |  |  |  |
| No | 29 (17.3 %) | 9 (4.9 %) | 2 (0.7 %) | 40 (6.1 %) | 0 (0.0 %) | 1 (0.8 %) | 1 (0.1 %) | 2 (0.2 %) |
| Yes | 139 (82.7 %) | 173 (95.1 %) | 302 (99.3 %) | 614 (93.9 %) | 59 (100.0 %) | 117 (99.2 %) | 922 (99.9 %) | 1098 (99.8 %) |
| e62 |  |  |  |  |  |  |  |  |
| No | 32 (19.0 %) | 9 (4.9 %) | 2 (0.7 %) | 43 (6.6 %) | 0 (0.0 %) | 0 (0.0 %) | 1 (0.1 %) | 1 (0.1 %) |
| Yes | 136 (81.0 %) | 173 (95.1 %) | 302 (99.3 %) | 611 (93.4 %) | 59 (100.0 %) | 118 (100.0 %) | 922 (99.9 %) | 1099 (99.9 %) |
| e7 |  |  |  |  |  |  |  |  |
| No | 119 (70.8 %) | 104 (57.1 %) | 72 (23.7 %) | 295 (45.1 %) | 34 (57.6 %) | 57 (48.3 %) | 132 (14.3 %) | 223 (20.3 %) |
| Yes | 49 (29.2 %) | 78 (42.9 %) | 232 (76.3 %) | 359 (54.9 %) | 25 (42.4 %) | 61 (51.7 %) | 791 (85.7 %) | 877 (79.7 %) |
| e8 |  |  |  |  |  |  |  |  |
| No | 80 (47.6 %) | 33 (18.1 %) | 14 (4.6 %) | 127 (19.4 %) | 6 (10.2 %) | 10 (8.5 %) | 12 (1.3 %) | 28 (2.5 %) |
| Yes | 88 (52.4 %) | 149 (81.9 %) | 290 (95.4 %) | 527 (80.6 %) | 53 (89.8 %) | 108 (91.5 %) | 911 (98.7 %) | 1072 (97.5 %) |
| e91 |  |  |  |  |  |  |  |  |
| No | 69 (41.1 %) | 30 (16.5 %) | 16 (5.3 %) | 115 (17.6 %) | 6 (10.2 %) | 4 (3.4 %) | 11 (1.2 %) | 21 (1.9 %) |
| Yes | 99 (58.9 %) | 152 (83.5 %) | 288 (94.7 %) | 539 (82.4 %) | 53 (89.8 %) | 114 (96.6 %) | 912 (98.8 %) | 1079 (98.1 %) |
| e92 |  |  |  |  |  |  |  |  |
| No | 63 (37.5 %) | 13 (7.1 %) | 5 (1.6 %) | 81 (12.4 %) | 6 (10.2 %) | 13 (11.0 %) | 9 (1.0 %) | 28 (2.5 %) |
| Yes | 105 (62.5 %) | 169 (92.9 %) | 299 (98.4 %) | 573 (87.6 %) | 53 (89.8 %) | 105 (89.0 %) | 914 (99.0 %) | 1072 (97.5 %) |
| e93 |  |  |  |  |  |  |  |  |
| No | 72 (42.9 %) | 25 (13.7 %) | 14 (4.6 %) | 111 (17.0 %) | 12 (20.3 %) | 25 (21.2 %) | 43 (4.7 %) | 80 (7.3 %) |
| Yes | 96 (57.1 %) | 157 (86.3 %) | 290 (95.4 %) | 543 (83.0 %) | 47 (79.7 %) | 93 (78.8 %) | 880 (95.3 %) | 1020 (92.7 %) |
| e10 |  |  |  |  |  |  |  |  |
| No | 84 (50.0 %) | 57 (31.3 %) | 27 (8.9 %) | 168 (25.7 %) | 12 (20.3 %) | 25 (21.2 %) | 27 (2.9 %) | 64 (5.8 %) |
| Yes | 84 (50.0 %) | 125 (68.7 %) | 277 (91.1 %) | 486 (74.3 %) | 47 (79.7 %) | 93 (78.8 %) | 896 (97.1 %) | 1036 (94.2 %) |
| e16 |  |  |  |  |  |  |  |  |
| No | 143 (85.1 %) | 105 (57.7 %) | 75 (24.7 %) | 323 (49.4 %) | 26 (44.1 %) | 41 (34.7 %) | 85 (9.2 %) | 152 (13.8 %) |
| Yes | 25 (14.9 %) | 77 (42.3 %) | 229 (75.3 %) | 331 (50.6 %) | 33 (55.9 %) | 77 (65.3 %) | 838 (90.8 %) | 948 (86.2 %) |
| f1 |  |  |  |  |  |  |  |  |
| No | 76 (45.2 %) | 132 (72.5 %) | 279 (91.8 %) | 487 (74.5 %) | 26 (44.1 %) | 69 (58.5 %) | 864 (93.6 %) | 959 (87.2 %) |
| Yes | 92 (54.8 %) | 50 (27.5 %) | 25 (8.2 %) | 167 (25.5 %) | 33 (55.9 %) | 49 (41.5 %) | 59 (6.4 %) | 141 (12.8 %) |
| f2 |  |  |  |  |  |  |  |  |
| No | 77 (45.8 %) | 127 (69.8 %) | 265 (87.2 %) | 469 (71.7 %) | 30 (50.8 %) | 66 (55.9 %) | 839 (90.9 %) | 935 (85.0 %) |
| Yes | 91 (54.2 %) | 55 (30.2 %) | 39 (12.8 %) | 185 (28.3 %) | 29 (49.2 %) | 52 (44.1 %) | 84 (9.1 %) | 165 (15.0 %) |
| f3 |  |  |  |  |  |  |  |  |
| No | 114 (67.9 %) | 151 (83.0 %) | 292 (96.1 %) | 557 (85.2 %) | 34 (57.6 %) | 67 (56.8 %) | 868 (94.0 %) | 969 (88.1 %) |
| Yes | 54 (32.1 %) | 31 (17.0 %) | 12 (3.9 %) | 97 (14.8 %) | 25 (42.4 %) | 51 (43.2 %) | 55 (6.0 %) | 131 (11.9 %) |
| f4 |  |  |  |  |  |  |  |  |
| No | 97 (57.7 %) | 162 (89.0 %) | 292 (96.1 %) | 551 (84.3 %) | 34 (57.6 %) | 81 (68.6 %) | 857 (92.8 %) | 972 (88.4 %) |
| Yes | 71 (42.3 %) | 20 (11.0 %) | 12 (3.9 %) | 103 (15.7 %) | 25 (42.4 %) | 37 (31.4 %) | 66 (7.2 %) | 128 (11.6 %) |
| f5 |  |  |  |  |  |  |  |  |
| No | 56 (33.3 %) | 140 (76.9 %) | 290 (95.4 %) | 486 (74.3 %) | 28 (47.5 %) | 81 (68.6 %) | 887 (96.1 %) | 996 (90.5 %) |
| Yes | 112 (66.7 %) | 42 (23.1 %) | 14 (4.6 %) | 168 (25.7 %) | 31 (52.5 %) | 37 (31.4 %) | 36 (3.9 %) | 104 (9.5 %) |
| f6 |  |  |  |  |  |  |  |  |
| No | 73 (43.5 %) | 141 (77.5 %) | 293 (96.4 %) | 507 (77.5 %) | 36 (61.0 %) | 93 (78.8 %) | 869 (94.1 %) | 998 (90.7 %) |
| Yes | 95 (56.5 %) | 41 (22.5 %) | 11 (3.6 %) | 147 (22.5 %) | 23 (39.0 %) | 25 (21.2 %) | 54 (5.9 %) | 102 (9.3 %) |
| f7 |  |  |  |  |  |  |  |  |
| No | 92 (54.8 %) | 136 (74.7 %) | 285 (93.8 %) | 513 (78.4 %) | 17 (28.8 %) | 28 (23.7 %) | 663 (71.8 %) | 708 (64.4 %) |
| Yes | 76 (45.2 %) | 46 (25.3 %) | 19 (6.2 %) | 141 (21.6 %) | 42 (71.2 %) | 90 (76.3 %) | 260 (28.2 %) | 392 (35.6 %) |
| f8 |  |  |  |  |  |  |  |  |
| No | 44 (26.2 %) | 80 (44.0 %) | 192 (63.2 %) | 316 (48.3 %) | 15 (25.4 %) | 7 (5.9 %) | 6 (0.7 %) | 28 (2.5 %) |
| Yes | 124 (73.8 %) | 102 (56.0 %) | 112 (36.8 %) | 338 (51.7 %) | 44 (74.6 %) | 111 (94.1 %) | 917 (99.3 %) | 1072 (97.5 %) |
| h1 |  |  |  |  |  |  |  |  |
| Mean (SD) | 2.3 (± 1.3) | 2.7 (± 1.3) | 3.4 (± 1.4) | 2.9 (± 1.4) | 2.4 (± 1.2) | 2.9 (± 1.1) | 3.4 (± 1.2) | 3.3 (± 1.2) |
| h2 |  |  |  |  |  |  |  |  |
| 1 | 74 (44.0 %) | 73 (40.1 %) | 98 (32.2 %) | 245 (37.5 %) | 44 (74.6 %) | 78 (66.1 %) | 574 (62.2 %) | 696 (63.3 %) |
| 2 | 6 (3.6 %) | 2 (1.1 %) | 4 (1.3 %) | 12 (1.8 %) | 0 (0.0 %) | 3 (2.5 %) | 8 (0.9 %) | 11 (1.0 %) |
| 3 | 0 (0.0 %) | 3 (1.6 %) | 4 (1.3 %) | 7 (1.1 %) | 2 (3.4 %) | 1 (0.8 %) | 3 (0.3 %) | 6 (0.5 %) |
| 4 | 13 (7.7 %) | 26 (14.3 %) | 39 (12.8 %) | 78 (11.9 %) | 7 (11.9 %) | 23 (19.5 %) | 186 (20.2 %) | 216 (19.6 %) |
| 5 | 25 (14.9 %) | 33 (18.1 %) | 92 (30.3 %) | 150 (22.9 %) | 5 (8.5 %) | 11 (9.3 %) | 148 (16.0 %) | 164 (14.9 %) |
| 6 | 33 (19.6 %) | 35 (19.2 %) | 59 (19.4 %) | 127 (19.4 %) | 0 (0.0 %) | 1 (0.8 %) | 3 (0.3 %) | 4 (0.4 %) |
| 7 | 8 (4.8 %) | 5 (2.7 %) | 2 (0.7 %) | 15 (2.3 %) | 0 (0.0 %) | 0 (0.0 %) | 0 (0.0 %) | 0 (0.0 %) |
| 8 | 2 (1.2 %) | 2 (1.1 %) | 1 (0.3 %) | 5 (0.8 %) | 1 (1.7 %) | 1 (0.8 %) | 0 (0.0 %) | 2 (0.2 %) |
| 9 | 7 (4.2 %) | 3 (1.6 %) | 5 (1.6 %) | 15 (2.3 %) | 0 (0.0 %) | 0 (0.0 %) | 1 (0.1 %) | 1 (0.1 %) |
| h3 |  |  |  |  |  |  |  |  |
| 1 | 3 (1.8 %) | 1 (0.5 %) | 2 (0.7 %) | 6 (0.9 %) | 0 (0.0 %) | 1 (0.8 %) | 5 (0.5 %) | 6 (0.5 %) |
| 2 | 50 (29.8 %) | 58 (31.9 %) | 136 (44.7 %) | 244 (37.3 %) | 46 (78.0 %) | 99 (83.9 %) | 798 (86.5 %) | 943 (85.7 %) |
| 3 | 2 (1.2 %) | 2 (1.1 %) | 0 (0.0 %) | 4 (0.6 %) | 0 (0.0 %) | 0 (0.0 %) | 0 (0.0 %) | 0 (0.0 %) |
| 4 | 1 (0.6 %) | 1 (0.5 %) | 4 (1.3 %) | 6 (0.9 %) | 0 (0.0 %) | 0 (0.0 %) | 4 (0.4 %) | 4 (0.4 %) |
| 5 | 110 (65.5 %) | 120 (65.9 %) | 161 (53.0 %) | 391 (59.8 %) | 13 (22.0 %) | 18 (15.3 %) | 116 (12.6 %) | 147 (13.4 %) |
| 7 | 2 (1.2 %) | 0 (0.0 %) | 1 (0.3 %) | 3 (0.5 %) | 0 (0.0 %) | 0 (0.0 %) | 0 (0.0 %) | 0 (0.0 %) |
| h61 |  |  |  |  |  |  |  |  |
| No | 78 (46.4 %) | 87 (47.8 %) | 117 (38.5 %) | 282 (43.1 %) | 15 (25.4 %) | 36 (30.5 %) | 247 (26.8 %) | 298 (27.1 %) |
| Yes | 90 (53.6 %) | 95 (52.2 %) | 187 (61.5 %) | 372 (56.9 %) | 44 (74.6 %) | 82 (69.5 %) | 676 (73.2 %) | 802 (72.9 %) |
| h62 |  |  |  |  |  |  |  |  |
| No | 133 (79.2 %) | 147 (80.8 %) | 246 (80.9 %) | 526 (80.4 %) | 39 (66.1 %) | 88 (74.6 %) | 743 (80.5 %) | 870 (79.1 %) |
| Yes | 35 (20.8 %) | 35 (19.2 %) | 58 (19.1 %) | 128 (19.6 %) | 20 (33.9 %) | 30 (25.4 %) | 180 (19.5 %) | 230 (20.9 %) |
| h63 |  |  |  |  |  |  |  |  |
| No | 125 (74.4 %) | 126 (69.2 %) | 211 (69.4 %) | 462 (70.6 %) | 26 (44.1 %) | 51 (43.2 %) | 506 (54.8 %) | 583 (53.0 %) |
| Yes | 43 (25.6 %) | 56 (30.8 %) | 93 (30.6 %) | 192 (29.4 %) | 33 (55.9 %) | 67 (56.8 %) | 417 (45.2 %) | 517 (47.0 %) |
| h64 |  |  |  |  |  |  |  |  |
| No | 138 (82.1 %) | 150 (82.4 %) | 257 (84.5 %) | 545 (83.3 %) | 48 (81.4 %) | 104 (88.1 %) | 860 (93.2 %) | 1012 (92.0 %) |
| Yes | 30 (17.9 %) | 32 (17.6 %) | 47 (15.5 %) | 109 (16.7 %) | 11 (18.6 %) | 14 (11.9 %) | 63 (6.8 %) | 88 (8.0 %) |
| h65 |  |  |  |  |  |  |  |  |
| No | 163 (97.0 %) | 176 (96.7 %) | 295 (97.0 %) | 634 (96.9 %) | 59 (100.0 %) | 117 (99.2 %) | 922 (99.9 %) | 1098 (99.8 %) |
| Yes | 5 (3.0 %) | 6 (3.3 %) | 9 (3.0 %) | 20 (3.1 %) | 0 (0.0 %) | 1 (0.8 %) | 1 (0.1 %) | 2 (0.2 %) |
| h66 |  |  |  |  |  |  |  |  |
| No | 168 (100.0 %) | 182 (100.0 %) | 296 (97.4 %) | 646 (98.8 %) | 59 (100.0 %) | 117 (99.2 %) | 919 (99.6 %) | 1095 (99.5 %) |
| Yes | 0 (0.0 %) | 0 (0.0 %) | 8 (2.6 %) | 8 (1.2 %) | 0 (0.0 %) | 1 (0.8 %) | 4 (0.4 %) | 5 (0.5 %) |
| h8 |  |  |  |  |  |  |  |  |
| Mean (SD) | 2.8 (± 2.3) | 2.8 (± 2.3) | 3.0 (± 2.5) | 2.9 (± 2.4) | 8.2 (± 27) | 3.3 (± 2.7) | 3.3 (± 7.7) | 3.6 (± 9.5) |
| h9 |  |  |  |  |  |  |  |  |
| Mean (SD) | 1.2 (± 0.44) | 1.2 (± 0.52) | 1.2 (± 0.47) | 1.2 (± 0.48) | 1.2 (± 0.56) | 1.4 (± 0.81) | 1.2 (± 0.64) | 1.2 (± 0.66) |
| h10 |  |  |  |  |  |  |  |  |
| 1 | 162 (96.4 %) | 164 (90.1 %) | 277 (91.1 %) | 603 (92.2 %) | 44 (74.6 %) | 95 (80.5 %) | 766 (83.0 %) | 905 (82.3 %) |
| 0 | 6 (3.6 %) | 18 (9.9 %) | 27 (8.9 %) | 51 (7.8 %) | 15 (25.4 %) | 23 (19.5 %) | 157 (17.0 %) | 195 (17.7 %) |
